# PixMed-Enhancer: An Efficient Approach for Medical Image Augmentation

**DOI:** 10.3390/bioengineering12030235

**Published:** 2025-02-26

**Authors:** M. J. Aashik Rasool, Akmalbek Abdusalomov, Alpamis Kutlimuratov, M. J. Akeel Ahamed, Sanjar Mirzakhalilov, Abror Shavkatovich Buriboev, Heung Seok Jeon

**Affiliations:** 1 Department of Computer Engineering, Gachon University, Sujeong-gu, Seongnam-si 13120, Republic of Koreabobomirzaevich@gmail.com (A.A.); abror1989@gachon.ac.kr (A.S.B.); 2 Department of Artificial Intelligence, Tashkent State University of Economics, Tashkent 100066, Uzbekistan; akmaljon@gachon.ac.kr (A.K.); mirzaxalilov86@tuit.uz (S.M.); 3 Department of Software Technology, Konkuk University, Chungju 27478, Republic of Korea

**Keywords:** medical image augmentation, image augmentation, conditional GAN, AI healthcare

## Abstract

AI-powered medical imaging faces persistent challenges, such as limited datasets, class imbalances, and high computational costs. To overcome these barriers, we introduce PixMed-Enhancer, a novel conditional GAN that integrates the ghost module into its encoder—a pioneering approach that achieves efficient feature extraction while significantly reducing the computational complexity without compromising the performance. Our method features a hybrid loss function, uniquely combining binary cross-entropy (BCE) and a Structural Similarity Index Measure (SSIM), to ensure pixel-level precision while enhancing the perceptual realism. Additionally, the use of conditional input masks offers unparalleled control over the generation of tumor features, marking a breakthrough in fine-grained dataset augmentation for segmentation and diagnostic tasks. Rigorous testing on diverse datasets establishes PixMed-Enhancer as a state-of-the-art solution, excelling in its realism, structural fidelity, and computational efficiency. PixMed-Enhancer establishes a robust foundation for real-world clinical applications in AI-driven medical imaging.

## 1. Introduction

In modern artificial intelligence (AI) healthcare, medical imaging plays a crucial role [1,2,3]. Specialists analyze and segment medical images to detect disorders and, in some instances, forecast how diseases may progress [4]. This approach also benefits precision medicine by enabling more tailored treatment plans based on individual patient characteristics. Medical image processing used to be performed manually by specialists [5]. However, due to the rapid development of imaging technology, many medical images are produced daily [2]. This influx of images demands considerable time for processing and necessitates specialized knowledge in the field. As a result, there is an increasing necessity to automate this process. The optimal way to achieve automation in medical image processing is by framing it as a deep neural network (DNN) challenge and addressing it by training a deep learning (DL) model [6]. To boost the performance of the DL model, it is essential to train it on a large and accurately labeled dataset. Moreover, when the objective is disease classification, it is crucial for the model to be trained on a balanced representation of each class with precise labeling [7]. In essence, data diversity is vital to enhancing the generalizability of the developed model; without it, the model is likely to exhibit a suboptimal performance [8,9].

Developing an effective DL model for medical image processing presents numerous challenges. One primary obstacle is the acquisition of adequate data, which is contingent on various factors, such as the ability to share data between healthcare organizations and researchers while upholding the confidentiality of patient information [10]. Another significant hurdle is the shortage of labeled data, which greatly impedes the creation of reliable image segmentation and classification models; manual annotation of medical images is often arduous and time-consuming and lacks consistency across different imaging modalities. Furthermore, many datasets exhibit imbalances [11], as the prevalence of disorders is typically much lower than that of non-diseased conditions, making the development of an automated diagnostic system considerably more complex. Consequently, researchers began to explore ways to enhance medical image datasets to develop effective classification or segmentation models. One strategy for enriching the original dataset is data augmentation, which encompasses the following: (1) fundamental image augmentation techniques (such as flipping, zoom, shear, rotation, cropping, and color adjustment) [12] and (2) deep learning methods (like Generative Adversarial Networks (GANs) and Neural Style Transfer) [12]. While basic augmentation techniques are limited in the volume of data they can produce and rely solely on the original dataset, deep learning methods, particularly GANs [13,14], can generate a much broader variety of data independently of the original dataset [15,16,17].

However, several critical factors dominate in the realm of medical image augmentation. Foremost among these are the precision of image reconstruction and an acute awareness of the hardware limitations, both of which are paramount in achieving groundbreaking results. Despite advancements, the current approaches struggle with persistent challenges, including blurred outputs, jagged artifacts, and noise-infused images. Moreover, these methods are computationally expensive, hindering their efficiency and practicality. To overcome these obstacles, we propose a conditional GAN approach that generates realistic images comparable to the ground truth with reduced computational requirements relative to those of existing methods. In the encoder component of our GAN, we incorporate the GhostNet module, which generates simplified "ghost" features by applying computationally inexpensive transformations to a subset of primary features. This enables the encoder to capture relevant details more efficiently, a critical improvement given that feature diversity significantly enhances the quality of GAN outputs.The contributions of this work can be outlined as follows:

- We incorporate the GhostNet module into the encoder component of our GAN, achieving substantial reductions in the computational requirements without compromising image quality. This integration allows for efficient feature extraction and enhances the model’s suitability for real-time applications in resource-constrained environments, marking a pioneering use of the ghost module in GAN architectures for medical purposes.
- We propose a novel combined loss function for optimizing the generator in adversarial training. This loss function merges the benefits of binary cross-entropy loss (BCE) and Structural Similarity Index Measure (SSIM) loss, offering a well-rounded objective for high-quality image generation. By combining BCE’s pixel-level accuracy with the SSIM’s focus on perceptual quality, this approach ensures both precise reconstruction and visually faithful outputs. The weighted combination of these losses enables the generator to optimize for structural similarity as well as pixel-level precision, overcoming the limitations of using a single loss function. Our combined loss function has demonstrated a superior performance, outperforming the traditional loss functions in both its quantitative metrics and visual quality, making it an innovative approach for generator training in adversarial models.
- The proposed model leverages conditional input masks to generate highly specific tumor images, facilitating precise control over the synthesis of pathological features. This capability is essential for augmenting datasets in tumor detection and segmentation tasks, thereby improving the diagnostic accuracy and operational workflows in clinical settings.
- Our method has been evaluated on diverse datasets, demonstrating enhanced realism and efficiency in generating synthetic tumor images. The practical applicability of our approach is further underscored by its potential to support AI-driven diagnostics and improve the robustness of medical imaging pipelines.
- A comprehensive evaluation was performed on a range of state-of-the-art models, including our proposed PixMed-Enhancer approach, to analyze their performance metrics.

The rest of this paper is structured as follows: Section 2 reviews related work on medical image augmentation methods. Section 3 describes the proposed architecture and outlines our augmentation strategy. Section 4 discusses an analysis of the performance metrics applied in this study. Finally, Section 5 provides the conclusions, highlighting limitations and future research directions.

## 2. Related Works

Due to the shortage of labeled image datasets, medical image augmentation has become more necessary in the process of developing strong deep learning models for healthcare applications [11,12]. In this section, a variety of medical image augmentation techniques, including traditional and advanced generative augmentation techniques, are discussed. And the shortcomings that our proposed method aims to address are highlighted in this section’s study of state-of-the-art GAN-based medical image synthesis.

### 2.1. Traditional Data Augmentation Techniques

Traditional methods for data augmentation have frequently been used to increase datasets and strengthen model robustness, especially in the medical field, where data scarcity is an issue. These traditional data augmentation methods can be broadly divided into geometric transformations and intensity-based adjustments.

#### 2.1.1. Geometric Transformations

Geometric transformations involve a range of methods that modify the characteristics of images while keeping their content unchanged. A few examples of geometric transformations are rotation (rotating images involves adjusting them at certain angles to mimic certain orientations), scaling (changes in scale involve adjusting the size of images to simulate differences in the sizes or distances of objects), flipping (when we flip images, we can mirror them either horizontally or vertically), and elastic deformations (simulating changes by non-linearly distorting images is known as elastic deformation) [18]. To increase the size of the training datasets and strengthen the robustness of machine learning models, traditional data augmentation methods, including elastic deformations and affine transformations (rotation, scaling, and skewing), have been widely employed [18].

One crucial method is to create elastic distortions that mimic real data variations. This technique has shown promising results, especially in applications like handwriting detection [18], as it creates new data points with modifications that maintain the basic structure of the original images. Ronneberger et al. have adapted similar techniques for use in medical imaging, utilizing elastic deformations in their U-Net architecture for biomedical image segmentation tasks [19]. These methods typically involve creating elastic distortions that mimic real-world data fluctuations. The images are initially changed using random displacement fields, and then they are smoothed using Gaussian filters to produce new data points with modifications that preserve the fundamental structure of the original images [18,19].

Perez and Wang [20] carried out an extensive study on the usefulness of different data augmentation methods in medical imaging. They discovered that integrating several augmentation techniques, such as geometric transformations, significantly enhanced the model’s performance on several medical imaging tasks.

#### 2.1.2. Intensity-Based Adjustments

The goal of intensity-based adjustments is to replicate changes in lighting, contrast, and other aspects of an image by modifying the pixel values of the image. Common methods include Brightness Adjustment (modifying the level of brightness of an image), Contrast Manipulation (modifying the intensity range of an image), Gamma Correction (the process of non-linearly adjusting the image intensity to replicate different imaging conditions), and Noise Injection (creating various types of noise to simulate defects in image acquisition).

#### 2.1.3. Limitations of the Traditional Techniques in Medical Imaging Augmentation

Limited complexity: The complex variations seen in medical imaging datasets are difficult for traditional data augmentation techniques to capture [21]. The model’s capacity to generalize across complex anatomical structures and pathological features is restricted by the frequent inability of geometric transformations and simple intensity modifications to produce enough varied training examples.

Potential for unrealistic transformations: According to Athalye and Arnaout, using traditional augmentation techniques requires considerable thought about domain-specific aspects. For example, flipping or rotating images is an example of a transformation that might change important parts of the data and possibly result in inaccurate interpretations. To make sure that augmentations do not destroy the integrity of the images or hide crucial anatomical information, the authors highlight that extensive knowledge of the medical context is essential [22].

Loss of critical information: Some augmentation methods carry the risk of eliminating or distorting crucial diagnostic characteristics, which could hinder the clinical usefulness of the images [22].

These traditional techniques can improve the model’s performance; they might still be unable to produce a variety of medical picture variations, such as tumor data, which require more specialized transformations specific to medical imaging scenarios. The produced transformations mostly rely on controlled distortion fields, which may not adequately represent the complex diversity present in medical image collections, especially when tasks like tumor diagnosis require high precision. Furthermore, elastic deformations may not be as well suited to medical image augmentation, as they are to basic image types, such as handwritten digits, which could result in a less than ideal performance when processing sensitive medical data. The unique difficulties in medical image augmentation, particularly for complicated conditions like tumors, necessitate more sophisticated approaches that can integrate domain-specific knowledge and more accurately replicate realistic variations in medical images, even though these techniques have proven effective in many fields.

As a result, more advanced methods, such as Generative Adversarial Networks (GANs), have acquired popularity due to their ability to produce synthetic data that are both high-quality and diverse, emulating real-world variations.

### 2.2. Advanced GAN Data Augmentation Techniques

Many of the drawbacks of the traditional approaches to medical image synthesis and augmentation have been addressed using Generative Adversarial Networks (GANs), which have become an effective method. Goodfellow et al. [23] introduced GANs, which are made up of two neural networks, a discriminator and a generator, that are trained concurrently via adversarial training.

#### 2.2.1. GANs in Medical Image Synthesis

In a variety of modalities, GANs have demonstrated exceptional potential to produce realistic medical images. The use of GANs to generate liver lesions in CT images was proven by Frid-Adar et al. [14], greatly enhancing the performance of lesion classification models. By creating synthetic samples that increased the diversity of the training dataset, their method addressed the problem of data scarcity in medical imaging.

Yi et al. [24] presented an in-depth review of GAN applications in medical imaging, highlighting their capacity for data synthesis and augmentation across many modalities, including MRI, CT, and X-ray. Their study demonstrates how versatile GANs are for problems like cross-modality synthesis and image denoising.

#### 2.2.2. Conditional GANs for Medical Imaging

As an extension of the original GAN framework, conditional GANs (cGANs) have proven very effective in medical imaging tasks because of their capacity to produce images that are conditioned on specified qualities. Since their introduction by Mirza and Osindero [25], cGANs have been extensively modified for use in medical imaging tasks.

To illustrate the potential of conditional generation in maintaining particular anatomical traits, Mahmood et al. [26] used cGANs for multi-organ nuclei segmentation in histopathology images. Given that our suggested method seeks to produce outputs that closely match the ground truth class, this strategy is very relevant.

For medical image synthesis, Liu et al. [27] created a cGAN-based model called MiSrc-GAN, which incorporated a progressive resolution generator to improve the image quality. A unique cGAN framework was introduced by Hamghalam and Simpson [28] in the field of brain tumor imaging with the goal of improving the tumor subregion contrast for better segmentation. Their method, which consists of two models (ESGAN and EnhGAN), shows how cGANs can produce synthetic images with great contrast and less inter-class overlap.

Wang et al. [29] have built an innovative cGAN model for chest X-ray image creation that can synthesize images from six different medical situations. By adding high-fidelity synthetic images to already existing datasets, this method seeks to improve the resilience of the machine learning models used in medical diagnosis.

In addition, LSGAN [30] substitutes a least squares loss function for the conventional binary cross-entropy loss. By promoting outputs that are closer to the actual data distribution, this change helps stabilize training, lessens the mode collapse, and enhances the quality of the generated images. However, LSGAN fails to produce clear outputs and tends to hallucinate. The summary of related work on GANs in medical imaging is shown in Table 1.

#### 2.2.3. Challenges and Limitations of GANs in Medical Imaging and Research Gaps

Despite the promising potential of GANs in medical imaging, several significant challenges and limitations persist:

##### Artifact Generation

One of the most critical issues in GAN-generated medical images is the occurrence of artifacts or unrealistic features. Bône et al. revealed that while GANs can produce realistic-looking medical images, they often fail to capture the full richness of medical datasets [31]. These artifacts can significantly compromise the diagnostic utility of the generated images, potentially leading to misinterpretations in clinical contexts [32].

##### Model Complexity and Efficiency

The complexity of medical imaging data poses significant challenges for GAN training and efficiency. Medical imaging datasets are often limited in size due to privacy concerns and acquisition costs, which can lead to mode collapse or overfitting in GANs [27]. Training GANs on high-resolution medical images requires substantial computational resources. Chen et al. addressed this by proposing a multilevel densely connected SR network (mDCSRN) for brain structural magnetic resonance imaging images, achieving a six-fold increase in speed compared to that of other models based on GANs [33]. Furthermore, the relationship between the dataset size, image complexity, and GAN performance is not straightforward. Wang et al. introduced a data-centric optimization method to create efficient GAN training for medical image synthesis, considering the distribution of the image complexities [29]. These factors collectively contribute to the challenge of balancing the model’s complexity with the computational efficiency in medical imaging applications of GANs.

##### Research Gaps and Proposed Solutions

The primary research gap that this study addresses is the need for a GAN-based approach that can generate high-quality medical images while simultaneously reducing artifacts, maintaining computational efficiency, and ensuring clinical relevance. Our proposed method aims to bridge this gap by incorporating a ghost module to efficiently capture deep features while reducing the computational complexity, utilizing a mask-based approach to generating outputs that closely resemble the ground truth class, minimizing artifacts, employing a combined loss function (BCE and the SSIM) to balance between the pixel-level accuracy and structural fidelity of the generated images, and addressing the trade-off between the model’s complexity and performance, making this approach more suitable for real-world clinical applications with resource constraints.

By focusing on these aspects, our proposed solution aims to advance the field of GAN-based medical image generation, addressing key limitations in the current approaches and moving towards more practical and clinically relevant implementations.

## 3. Overview of the Proposed Method

In generative AI, GANs plays a prominent role. GANs aim to map a random noise vector *V* to generate an output image $I_{out}$ [34]. In terms of conditional GANs, they learn to map an observed image $I_{input}$ and a random noise vector *V* to an output $I_{out}$, represented as $C_{GAN}:\left\{I_{input},V\right\}\to I_{out}$. The generator $C_{GAN}$ is trained to produce outputs that the adversarially trained discriminator *D* cannot distinguish from real images. The discriminator *D*, in turn, is optimized to excel at identifying the generator’s synthetic outputs as fake.

However, this approach can produce artifacts or hallucinations that are reflected in the generated output [35]. Medical images need to be crisp with minimal distortion and precisely depict the original classes while additionally taking the model’s complexity into account. Complex models in medical device deployment might reveal challenges due to the resource limits, longer inference times, and energy efficiency. As a result, an appropriate ratio of the model complexity to the image quality and computing efficiency is required to provide a consistent and effective performance in real-world clinical situations.

### 3.1. The Proposed Approach

To address these challenges, we propose a GAN-based approach that leverages masks of medical images to generate outputs that closely resemble the ground truth class.

#### 3.1.1. The Ghost Module

**Figure 1:**
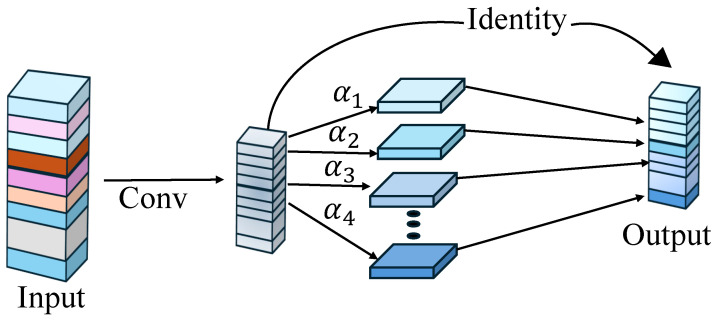
Overview of ghost module.

The ghost module [36] is a cost-effective neural network component that maintains or improves the performance. It works by creating ghost feature maps, which are lightweight and serve as resemblant approximations of the actual features. The ghost module begins by extracting features using typical convolution techniques, followed by a lightweight procedure that generates new feature maps from the original ones, thereby increasing the feature set while reducing the computing strain. This method enables the network to capture rich feature representations with fewer parameters, lowering the memory use and increasing the processing performance, making it especially helpful in resource-constrained contexts or when dealing with huge datasets. Using this strategy, networks may attain great efficiency while maintaining accuracy. An overview of the ghost module is explained in Figure 1.

The operation starts with an input tensor, which represents the feature maps retrieved from the previous layer of the neural network. A conventional convolution operation is applied to the input features, resulting in a series of intermediate feature maps. Instead of generating all of the output features using extremely costly traditional convolutions, the ghost module provides extra feature maps (denoted by $\alpha_{1},\alpha_{2},\alpha_{3},\alpha_{4}$, etc.) using linear operations, such as transformations or filters, which are significantly more efficient. Furthermore, some characteristics from the convolution process are transferred directly via identity mapping, which serves as the foundation for the final output. The final output feature maps are then created by integrating the lightweight ghost features with the identity-mapped features, resulting in computational efficiency while maintaining representational quality. Incorporating this module into the encoder part of our proposed architecture helps capture the deep features efficiently and accurately, which is very beneficial for passing information to the decoder part with reduced noise.

#### 3.1.2. Our Proposed Framework

**Figure 2:**
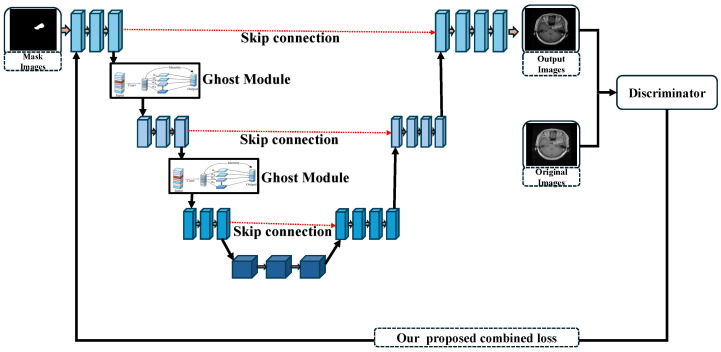
Proposed architecture of the PixMed-Enhancer. The red dotted line indicates a skip connection, while the solid lines represent connections between each convolutional layer.

The proposed framework employs a modified encoder architecture meticulously crafted to extract intricate and meaningful features from the input mask images. Central to this encoder is the integration of a connected ghost module, a breakthrough design that not only captures rich, deep features but also achieves remarkable reductions in the computational complexity. This innovative synergy ensures that the framework delivers a superior accuracy without compromising on efficiency, making it a robust solution for high-performance applications. The comprehensive design of the proposed approach is presented in Figure 2. The generator output is iteratively processed by the generator network, refining it in each iteration. The final result is then assessed using a modified loss function that is specific to our system. This tailored loss computation accurately reflects the differences between the generated and desired outputs. The computed loss value is then passed back to the generator, allowing for more accurate parameter modifications and increasing its ability to provide outputs that are closer to the ground truth.

#### 3.1.3. A Modified Loss Function for the Generator

To optimize the performance of the proposed model, a combined loss function $L_{\mathrm{combined}}$ is employed, which integrates binary cross-entropy (BCE) loss [37] and Structural Similarity Index Measure (SSIM) loss [38]. The BCE loss is calculated as follows:

(1) $$L_{\mathrm{BCE}}=-\frac{1}{N}\sum_{i=1}^{N}\left[y_{i}\cdot \log\left({\hat{y}}_{i}\right)+\left(1-y_{i}\right)\cdot \log\left(1-{\hat{y}}_{i}\right)\right],$$

where $y_{i}$ and ${\hat{y}}_{i}$ represent the true and predicted binary values, respectively, and *N* is the number of samples. The SSIM loss is derived from the Structural Similarity Index (SSIM), which measures the structural similarity between the predicted and ground truth data, ensuring the perceptual quality of the predictions. The combined loss function is then formulated as follows:

(2) $$L_{\mathrm{combined}}=L_{\mathrm{BCE}}+L_{\mathrm{SSIM}},$$

where $L_{\mathrm{SSIM}}=1-\mathrm{SSIM}\left(y_{\mathrm{true}},y_{\mathrm{pred}}\right)$, ensuring that minimizing the combined loss simultaneously reduces the pixel-level errors and enhances the structural fidelity of the predictions. This synergistic approach balances between the accuracy and perceptual quality, leading to a better overall model performance.

## 4. The Materials and Experimental Setup

### 4.1. The Dataset

**Figure 3:**
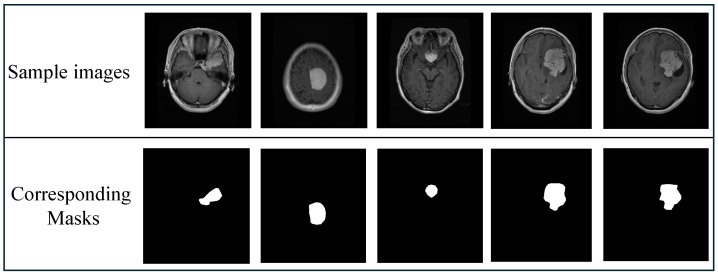
Representative examples of brain tumor images paired with their corresponding masks.

In this study, we leveraged the extensively utilized 2D brain tumor segmentation dataset [39], publicly available on Kaggle, which serves as a benchmark in medical imaging research. This dataset comprises an impressive collection of 3,064 images, each paired with its corresponding mask images. Figure 3 showcases a few representative examples from this dataset, highlighting the diversity and complexity of the dataset’s images.

### 4.2. Data Preprocessing

Initially, the data preparation consisted of three steps: scaling, cropping, and normalization. The collection originally included evenly sized images (256 × 256 × 3). First, the images were expanded to a larger dimension (286 × 286 × 3) to facilitate cropping. The images were then randomly cropped to their original size of 256 × 256 × 3, providing spatial variation.

The final stage in the preparation pipeline was normalization, which involved scaling the pixel values of the images to the range of [−1, 1]. This technique helped to limit the impact of illumination fluctuations throughout the dataset, guaranteeing consistency in the input data and increasing the model’s resilience. After normalization, the dataset was separated into three subsets, training, validation, and testing, at ratios of 0.8, 0.1, and 0.1, respectively. The data splitting was conducted swiftly and reliably using the split-folder library.

### 4.3. The Experimental Setup

This study was conducted using the Keras 3.8.0 framework within the Colab Pro environment, leveraging its advanced features. The experimental procedure encompassed several stages, including training, validation, and testing, with all being executed on a Tesla T4 GPU. Keras was selected due to its extensive support for deep learning applications and its intuitive interface, which facilitated efficient and streamlined model development. This choice granted access to a comprehensive library of prebuilt neural network architectures, optimization algorithms, and evaluation metrics. The networks developed in this study were trained with a batch size of 64 and a learning rate of 0.001, and the training process was carried out over 400 epochs utilizing the Adam optimizer exponential decay rate for $beta_{1}=0.5$.

## 5. The Experiment and Result Analysis

The effectiveness of the proposed approach was evaluated and benchmarked against state-of-the-art GAN-based augmentation techniques. Additionally, its applicability to various medical image augmentation tasks was confirmed through comprehensive and rigorous experiments.

### 5.1. Result Analysis

**Figure 4:**
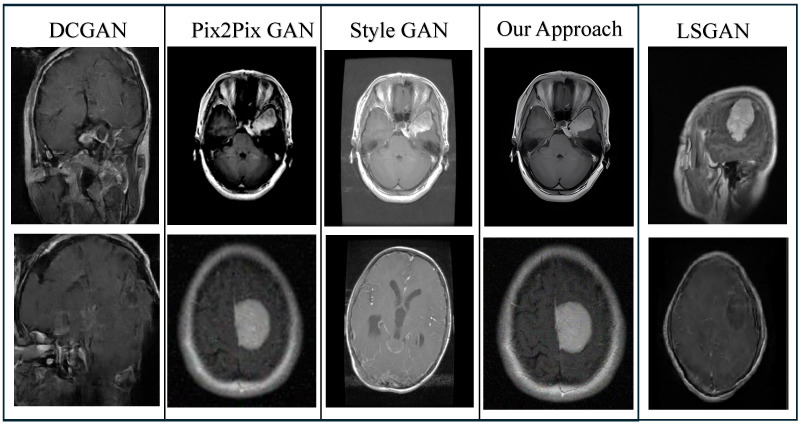
Comparative analysis of our approach for generated images with state-of-the-art (SOTA) methods.

Figure 4 illustrates a comprehensive comparison between our proposed approach and state-of-the-art (SOTA) image generation methods. Notably, the DC-GAN [40] and LSGAN [30] approaches exhibit significant limitations, producing highly unrealistic and heavily distorted images. In stark contrast, our approach consistently outperforms both pix2pix GAN [41] and StyleGAN [42], delivering visually superior and more coherent images with remarkable attention to detail and fidelity. This highlights the robustness and advanced capabilities of our method in generating realistic images compared to those of existing SOTA techniques.

To further evaluate the effectiveness of our proposed method, we utilized the Brain Tumor Segmentation dataset [39] and the Kaggle Brain Tumor Classification dataset [43] and trained the DenseNet201 [44] model to classify brain tumors. Compared to the images generated by the state-of-the-art frameworks, our approach not only achieved greater visual coherence but also yielded images with enhanced interpretability, thereby increasing their usefulness for downstream analytical tasks. These findings indicate that our method not only surpasses the current benchmarks in terms of image quality and practical application but also holds promise for advancing the state of the art in medical image generation and analysis. The results are presented in Table 2. Here, we generated 100 output images and conducted a classification comparison.

**Figure 5:**
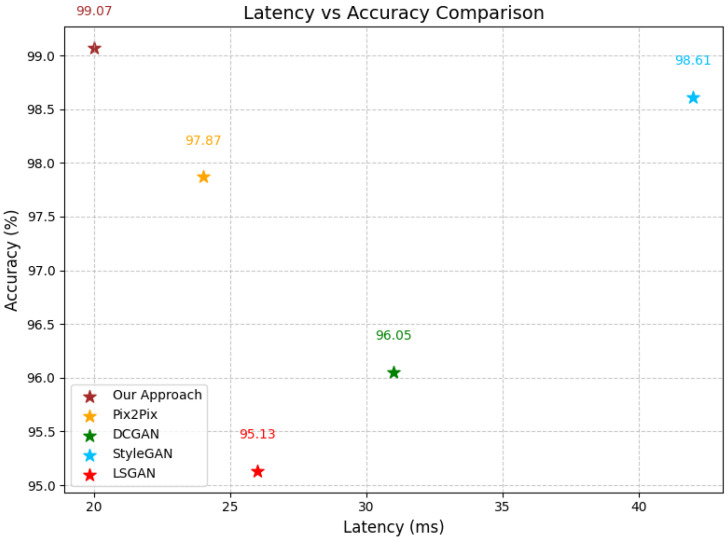
Comparative analysis of latency and accuracy between our approach and state-of-the-art (SOTA) methods.

In addition, to compare this approach in terms of its latency and efficiency, we presented a comparison of the latency of our approach with that of the other methods, as shown in Figure 5. Our approach demonstrates unparalleled simplicity and efficiency, outperforming the other state-of-the-art methods with significantly lower complexity and higher performance metrics. Additionally, we conducted a comparison of the memory usage of each approach and the structural similarity of the generated images to the ground truth images using the SSIM [45,46], and the results are illustrated in Table 3.

Furthermore, as part of an ablation study, we conducted the experiment without the ghost module, derived the outputs, and used the DenseNet201 model to classify brain tumors. The results are demonstrated in Table 4.

### 5.2. A Comparison of the Loss Function

**Figure 6:**
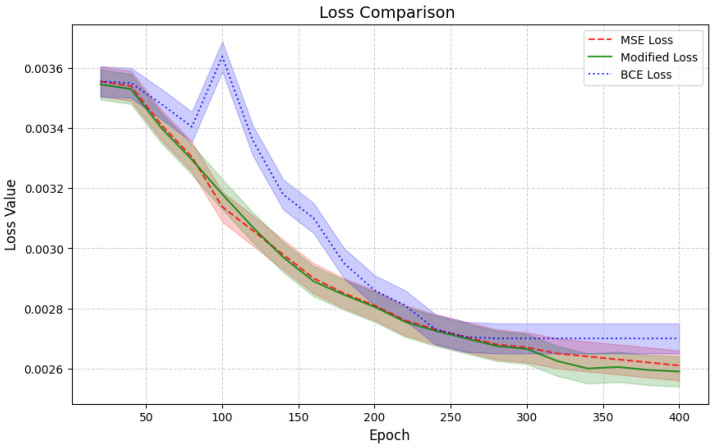
Comparison of convergence behavior and final performance between BCE, MSE, and our proposed loss function.

In addition, we conducted experiments comparing our proposed loss function against the BCE and MSE loss functions. In Figure 6, the blue line represents the BCE loss, the red line represents the MSE loss, and the green line represents our proposed modified loss function.

As illustrated, while the BCE loss converges, it ultimately stabilizes at a slightly higher value compared to that of both the MSE and our proposed approach. Notably, our modified loss function consistently outperforms BCE and MSE, achieving a significantly better performance in terms of the image quality and overall model optimization.

### 5.3. Discussion and Limitations

Our proposed approach also shows improvements over the current state-of-the-art methods in the generation of realistic medical images, especially for brain tumor classification. The application of connected ghost modules in the encoder architecture has been seen to be beneficial in identifying essential features while at the same time reducing the computational power needed. This innovation also provides a better performance in terms of accuracy as compared to that of the other methods while at the same time maintaining efficiency, making it suitable for high-performance medical imaging applications. When compared with DC-GAN, pix2pix GAN, and StyleGAN, our method is found to produce images with better visual coherence and quality. Not only do the generated images look more real but they are also easier to interpret, which is important for any subsequent analytical tasks in the context of medical imaging. The proposed approach gave the best classification performance of 99.07% and the lowest loss of 0.0458 when a DenseNet201 model was used on the Kaggle Brain Tumor Classification dataset. It outperforms other GAN models such as Pix2pix, DCGAN, and StyleGAN as well. The newly proposed loss function, which is a combination of binary cross-entropy (BCE) and Structural Similarity Index Measure (SSIM), has a better convergence than that of the pure BCE and MSE loss functions. This approach is effective in combining the accuracy of the pixel values with the structural correctness, thus enhancing the overall performance of the model. However, there are some drawbacks to this approach.

#### Limitations

The performance of our model has mainly been tested on brain tumor images, necessitating further assessment on other types of medical images and diseases. Although our approach demonstrates an improved efficiency compared to that of the current state-of-the-art methods, the training process remains computationally demanding, potentially limiting its use in resource-constrained settings. As with any AI-generated medical imagery, there are potential ethical implications regarding the use of synthetic data in clinical decision-making processes that need to be carefully addressed. The long-term stability and reliability of the generated images in clinical applications require further longitudinal studies. While our model shows impressive results on the given dataset, its performance on unseen data or different medical imaging modalities needs further validation.

Future work should focus on addressing these limitations, expanding the applications to diverse medical imaging domains, and conducting more extensive clinical validations to ensure the robustness and reliability of the proposed method in real-world medical scenarios.

## 6. Conclusions

In conclusion, our proposed method for medical image generation shows clear improvements over the existing techniques. By utilizing connected ghost modules and a modified loss function, we have created an approach that produces high-quality brain tumor images efficiently. The ghost modules allow for effective feature extraction while reducing the computational complexity, striking a balance between accuracy and efficiency. When compared to other GAN-based methods such as DC-GAN, pix2pix GAN, and StyleGAN, our approach consistently generates more realistic and coherent images. These improvements in image quality translate to a better performance in downstream tasks, as demonstrated by the classification results using a DenseNet201 model on the Kaggle Brain Tumor Classification dataset. The combined loss function, incorporating both binary cross-entropy and the Structural Similarity Index Measure, demonstrates better convergence behavior than that of standalone loss functions. This contributes to the overall effectiveness of our method in generating high-quality medical images. Our approach also demonstrates a lower latency while maintaining accuracy, suggesting its potential for real-time applications in medical imaging. These results indicate that our method could contribute to advancements in medical image generation and analysis.

In future, it will be important to address the limitations discussed earlier and to test this approach across a wider range of medical imaging applications. Further clinical validation will be necessary to fully assess its potential impact in real-world medical scenarios.

## Figures and Tables

**Table 1:** Summary of related work on GANs in medical imaging.

Study	Application	Explanation of Study
Perez and Wang (2017) [20]	Data Augmentation	Conducted a review of various data augmentation techniques and showed that integrating multiple augmentation methods significantly improves the model performance. However, these methods may fail to capture all real-world variations.
Frid-Adar et al. (2018) [14]	Liver Lesion Generation	Utilized GANs to synthesize liver lesions for enhancing training datasets in medical imaging tasks. Although promising, the synthetic images may lack clinical relevance or accuracy.
Hamghalam and Simpson (2021) [28]	Brain Tumor Imaging	Developed ESGAN and EnhGAN models to improve the contrast in tumor subregions. These models achieved enhanced segmentation with a reduced inter-class overlap but exhibited variability in their performance across different tumor types and imaging conditions.
Wang et al. (2022) [29]	Chest X-ray Image Synthesis	Proposed a cGAN model for synthesizing chest X-ray images across multiple conditions, improving diagnostic model resilience by augmenting datasets with high-fidelity images. However, there is a risk of overfitting to synthetic data rather than real-world variability.
Bône et al. (2021) [31]	Evaluation of GAN-Generated Images	Investigated artifacts in GAN-generated medical images, finding that these artifacts can compromise the diagnostic utility and lead to potential misinterpretations. The extent of the artifacts varies across different GAN architectures and configurations.

**Table 2:** Comparison of classification results based on accuracy, loss, and recall.

Dataset	GAN Model	Accuracy (%)	Loss	Recall
	Pix2pix [41]	97.87	0.0751	0.97
	DCGAN [40]	96.05	0.0983	0.95
[43]	StyleGAN [42]	98.61	0.0554	0.97
	LSGAN [30]	95.13	0.0882	0.95
	Our Approach	99.07	0.0458	0.98
	Pix2pix [41]	87.67	0.1151	0.87
	DCGAN [40]	85.03	0.1243	0.84
[39]	StyleGAN [42]	88.54	0.1003	0.88
	LSGAN [30]	84.32	0.1681	0.83
	Our Approach	89.58	0.0958	0.89

**Table 3:** Comparison of the memory usage and SSIM for the [43] dataset.

Approach	Memory Usage	SSIM
Pix2Pix [41]	54.31 MB	0.721
DCGAN [40]	460 MB	0.682
StyleGAN [42]	3.4 GB	0.736
LSGAN [30]	568 MB	0.673
Our approach	52.44 MB	0.742

**Table 4:** Results of ablation study on the impact of the ghost module.

Dataset	GAN Model	Accuracy (%)	Loss	Recall
	Our approach without the ghost module	96.57	0.0953	0.96
[43]	Our approach	99.07	0.0458	0.98
	Our approach without the ghost module	88.62	0.1051	0.88
[39]	Our approach	89.58	0.0958	0.89

## Data Availability

The datasets used and/or analyzed during the current study are available from the corresponding author upon reasonable request.
